# Management standards and burnout among surgeons in the United Kingdom

**DOI:** 10.1093/occmed/kqad102

**Published:** 2023-10-06

**Authors:** J Houdmont, P Daliya, A Adiamah, E Theophilidou, J Hassard, D N Lobo, East Midlands Surgical Academic Network (EMSAN) Burnout Study Group, Jamil Ahmed, Victor Babu, Daryll Baker, David Bartlett, Ian Beckingham, Imran Bhatti, Adam Brooks, Steven Brown, Josh Burke, Hannah Byrne, Ian Chetter, Hannah Cook, James Coulston, Lucinda Cruddas, Richard Dias, Frank Dor, Mukul Dube, Katherine Grant, John Hammond, Rachel Hargest, Theophilus Joachim, Annie Joseph, Naveed Kara, Dimitrios Karavias, Sita Kotecha, Anisa Kushairi, Roshan Lal, Kit Lam, Irwin Lasrado, Rachel Lee, Gurminder Mann, George Mannu, Charles Maxwell-Armstrong, James McCaslin, Frank McDermot, Andrew Miller, Sarah Miller, Jenna Morgan, Sandip Nandhra, Sangara Narayanasamy, Patrick O’Brien, Laura Parry, Kizzie Peters, Marina Pitsika, Emmanouil Psaltis, Kevin Sargen, Panchali Sarmah, Murali Shyamsundar, Chinnappa Reddy, Katie Rollins, Farah Roslan, Joseph Shalhoub, Matt Stanislas, Benjamin Tan, Nilanjana Tewari, Pradeep Thomas, Tony Thomas, Jim Tiernan, Giles Toogood, Karl Trimble, Peter Vaughan-Shaw, Luke Wheldon, Steven White, Tim White, Imeshi Wijetunga, Michael Wilson, Rebecca Winterborn, Lynda Wyld, Lora Young

**Affiliations:** School of Medicine, University of Nottingham, Nottingham NG8 1BB, UK; East Midlands Surgical Academic Network, Queen’s Medical Centre, Nottingham NG7 2UH, UK; Nottingham Digestive Diseases Centre and National Institute for Health Research (NIHR) Nottingham Biomedical Research Centre, Nottingham University Hospitals and University of Nottingham, Queen’s Medical Centre, Nottingham NG7 2UH, UK; East Midlands Surgical Academic Network, Queen’s Medical Centre, Nottingham NG7 2UH, UK; Nottingham Digestive Diseases Centre and National Institute for Health Research (NIHR) Nottingham Biomedical Research Centre, Nottingham University Hospitals and University of Nottingham, Queen’s Medical Centre, Nottingham NG7 2UH, UK; East Midlands Surgical Academic Network, Queen’s Medical Centre, Nottingham NG7 2UH, UK; Nottingham Digestive Diseases Centre and National Institute for Health Research (NIHR) Nottingham Biomedical Research Centre, Nottingham University Hospitals and University of Nottingham, Queen’s Medical Centre, Nottingham NG7 2UH, UK; School of Medicine, University of Nottingham, Nottingham NG8 1BB, UK; Management School, Queen’s University of Belfast, Belfast, BT9 5EE, UK; School of Medicine, University of Nottingham, Nottingham NG8 1BB, UK; East Midlands Surgical Academic Network, Queen’s Medical Centre, Nottingham NG7 2UH, UK; Nottingham Digestive Diseases Centre and National Institute for Health Research (NIHR) Nottingham Biomedical Research Centre, Nottingham University Hospitals and University of Nottingham, Queen’s Medical Centre, Nottingham NG7 2UH, UK; MRC-Versus Arthritis Centre for Musculoskeletal Ageing Research, School of Life Sciences, University of Nottingham, Queen’s Medical Centre, Nottingham NG7 2UH, UK

## Abstract

**Background:**

Burnout arising from chronic work-related stress is endemic among surgeons in the UK. Identification of contributory and modifiable psychosocial work characteristics could inform risk reduction activities.

**Aims:**

We aimed to assess the extent to which surgeons’ psychosocial working conditions met aspirational Management Standards delineated by the UK Health and Safety Executive, draw comparisons with national general workforce benchmarks and explore associations with burnout.

**Methods:**

Surgeons (*N* = 536) completed the Management Standards Indicator Tool and a single-item measure of burnout. Descriptive data were computed for each Standard, independent *t*-tests were used to examine differences between trainees and consultants, and hierarchical linear regression was applied to explore relations between psychosocial work environment quality and burnout.

**Results:**

Psychosocial work environment quality fell short of each Management Standard. Trainee surgeons (*n* = 214) reported significantly poorer psychosocial working conditions than consultant surgeons (*n* = 322) on the *control*, *peer support* and *change* Standards. When compared with UK workforce benchmarks, trainees’ psychosocial working conditions fell below the 10th percentile on four Standards and below the 50th percentile on the remainder. Consultant surgeons were below the 50th percentile on five of the seven Standards. Psychosocial working conditions accounted for 35% of the variance in burnout over that accounted for by socio- and occupational-demographic characteristics.

**Conclusions:**

Surgeons’ psychosocial working conditions were poor in comparison with benchmark data and associated with burnout. These findings suggest that risk management activities based on the Management Standards approach involving modification of psychosocial working conditions would help to reduce burnout in this population.

## Teaser

We examined the extent to which surgeons’ psychosocial working conditions achieved the Health and Safety Executive’s Management Standards quality targets and linked with burnout. Psychosocial work environment quality fell short of each Management Standard and in most cases below the national general workforce average. Problematic *job demands* (trainees and consultants), *managerial support* (trainees) and *control* (consultants) were most strongly linked to burnout. Risk management centred on these characteristics would likely help ameliorate burnout among surgeons.

**Key learning points**

**What is already known about this subject:**

- Burnout arising from chronic work-related stress is endemic among surgeons in the UK.
- A small body of mostly qualitative, small-scale or specialty-specific research has implicated a host of psychosocial working conditions found in the context (as opposed to clinical content) of work in the development of surgeon burnout.

**What this study adds:**

- This study builds on the existing evidence base by quantitatively examining the psychosocial working conditions experienced by a large sample of trainee and consultant surgeons across specialties using the UK Health and Safety Executive Management Standards framework.
- Psychosocial work environment quality fell short of each aspirational Management Standard; when compared with UK workforce benchmarks, trainee surgeons’ psychosocial working conditions fell below the 10th percentile on four Standards and below the 50th percentile on the remainder. Consultant surgeons were below the 50th percentile on five Standards.
- Psychosocial working conditions accounted for 35% of the variance in burnout over that accounted for by socio- and occupational-demographic characteristics.

**What impact this may have on practice or policy:**

- Our findings suggest that risk reduction initiatives involving modification of problematic *job demands* (trainee and consultant surgeons), *managerial support* (trainees) and *control* (consultants) would be particularly helpful in the amelioration of burnout in the surgical population.
- Healthcare organizations that adopt a risk management approach for dealing with psychosocial risk stand to reap the benefits of a healthier and more sustainable surgical workforce.

## Introduction

Psychosocial working conditions, ‘those aspects of work design and the organization and management of work, and their social and organizational contexts, which have the potential for causing psychological, social, or physical harm’ [1] have implications for the career longevity of surgeons. A 2021 survey of urological surgeons in Britain found that among those who had retired before 60 years of age, more than half cited a *bad workplace*, *work intensity* and *hospital management* as factors contributing to their decision to retire, while one-third cited *bad colleagues*. Among consultants still working, more than half of those with 11–15 years in the role reported *bad workplace* and *hospital management*, and two-fifths reported *bad colleagues*, as reasons for wishing to curtail their career [2].

In response to concerns about the sustainability of the surgical workforce in the UK, researchers have begun to investigate surgeons’ psychosocial work environment quality and propose modifications to help support lengthy and satisfying careers. Much of this research has been conducted through the lens of burnout, which is itself the most common reason given for considering exiting surgical training [3] and endemic across the profession [4]. The body of mostly qualitative, small-scale or specialty-specific research has implicated a host of psychosocial working conditions found in the context (as opposed to clinical content) of work in the development of burnout. Factors include excessive administrative workload, overall work volume, lack of institutional resources, interpersonal conflict, demand–resource imbalance, work–life imbalance, work-related pressure, lack of autonomy, and poor hospital and National Health Service management [5–8].

In the current study, our intention is to build on the existing evidence base by quantitatively examining the psychosocial working conditions experienced by a large sample of trainee and consultant surgeons across specialties using the Management Standards framework [9] delineated by the UK government through the agency of the Health and Safety Executive (HSE). The Management Standards approach was introduced to support organizations in the fulfilment of their duties under workplace health and safety legislation to minimize and where feasible eliminate exposure to potentially harmful psychosocial working conditions while taking steps to promote health and well-being by optimizing psychosocial work environment quality. The Standards pertain to seven key psychosocial work characteristics that if not properly managed can lead to harm (*demands*, *control*, *managerial support*, *peer support*, *relationships*, *role* and *change*), and for each there exists a description of the Standard and outline of what should be happening in the organization if the Standard is met. To enable organizations to assess the extent to which they meet the aspirational Management Standards, monitor improvements over time, and benchmark against the general UK workforce, the HSE published the Management Standards Indicator Tool (MSIT). The MSIT is a freely available (https://www.hse.gov.uk/stress/standards/downloads.htm), self-report survey instrument that assesses psychosocial work environment quality over the preceding 6-month period across the seven Management Standards. It has been used with a wide variety of occupational groups [10–15]. To date, its application to surgeons is limited to a single small-scale survey that utilized a subset of items from the *demands* and *managerial support* domains [16]. There remains a lacuna concerning the extent to which the psychosocial working conditions of surgeons working in the UK meet the aspirational targets set out in the Management Standards and how these might be linked to burnout. Greater knowledge on the nature and implications of surgeons’ psychosocial working conditions could usefully inform interventions to tackle burnout in the profession and enhance career sustainability. Thus, in a sample of surgeons in the UK, the aims of this study were to (i) assess the extent to which psychosocial working conditions measured using the MSIT achieved aspirational targets delineated in the Management Standards, (ii) compare MSIT scores to UK general workforce benchmarks and (iii) examine the strength and direction of associations between MSIT scores and burnout.

## Methods

Trainee and consultant surgeons working in the UK completed a self-report online survey between January and March 2021. The survey was piloted prior to full administration, resulting in a small number of amendments to aid clarity. The survey was promoted via social media (Twitter, Facebook and LinkedIn), regional research collaboratives, hospital group e-mails, posters and personal contact. The study was approved by the University of Nottingham Faculty of Medicine and Health Sciences Research Ethics Committee (FHMS 485-2002). The analyses reported herein refer to one element of the survey; other findings have been reported separately [4,17].

Psychosocial work environment quality was assessed using the 25-item version of the MSIT that was developed by deleting redundant items from the original 35-item measure [18]. The psychometric properties of the abridged MSIT have been demonstrated [18–21], and it is commonly used where an imperative exists for brevity in survey length [10]. Responses to the first 15 items are given on a five-point scale ranging from *never* (1) to *always* (5), with negatively framed items (e.g. ‘I have unachievable deadlines’) reverse scored so that low scores indicate poor psychosocial working conditions. Responses to the remaining items are given on a five-point scale of *strongly disagree* (1) to *strongly agree* (5), with negatively framed items (e.g. ‘I am subject to bullying at work’) reverse scored. A mean score is generated for each of the seven Management Standards: *demands*, *control*, *managerial support*, *peer support*, *relationships*, *role* and *change*. For each domain, a score of 1 indicates poor psychosocial work environment quality while 5 indicates that the Standard is being met. Benchmark data are available for the general UK workforce [18].

To assess burnout, we used a single-item measure developed for application with doctors [22] that has been validated among primary care and specialist doctors in the USA [23]. Research involving UK surgeons has indicated that this measure has high sensitivity in relation to the emotional exhaustion dimension of the ‘gold standard’ Maslach Burnout Inventory [17], and therefore offers a useful brief measure of that construct while minimizing the time required for survey completion. The stem question, ‘Overall, based on your definition of burnout, how would you rate your level of burnout?’ is followed by five response options: *I enjoy my work. I have no symptoms of burnout* (1), *Occasionally, I am under stress, and I don’t always have as much energy as I once did, but I don’t feel burned out* (2), *I am definitely burning out and have one or more symptoms of burnout, such as physical and emotional exhaustion* (3), *The symptoms of burnout that I’m experiencing won’t go away. I think about frustration at work a lot* (4), and *I feel completely burned out and often wonder if I can go on. I am at the point where I may need some changes or may need to seek some sort of help* (5). A score of ≥3 is used to identify burnout.

Separate analyses were conducted for trainee surgeons and consultant surgeons owing to previous research having identified contrasting burnout profiles in these groups [4]. To address the first two aims of the study, descriptive statistics (means and standard deviations) were calculated for each Management Standard and compared to UK general workforce benchmarks [18]. The benchmark dataset contains responses on the 25-item MSIT from 67 347 workers across 137 organizations, 36 of which were health sector organizations (mostly NHS trusts). We applied unrelated *t*-tests to identify significant differences between trainee and consultant surgeons on psychosocial work environment quality. For the third aim of the study, Pearson’s correlations were used to establish the strength and direction of relations between psychosocial work environment quality and burnout. Following pre-analysis checks, hierarchical linear regression was used to evaluate the relative contribution of the Management Standards to burnout after controlling for age and sex (and grade in the trainee analysis). We also calculated the proportion of respondents reporting burnout.

## Results

A total of 621 surveys containing responses were submitted, with analyses conducted for respondents who completed the MSIT and burnout measure and indicated they were trainee surgeon (CT1/ST1 to ST8) (*n* = 214) or consultant surgeon (*n* = 322). Participants’ socio- and occupational-demographic characteristics are displayed in Table 1. Most trainees (85%, 182/214) and consultants (81%, 260/322) worked in England, with the remaining responses drawn from Wales, Scotland and Northern Ireland. General and vascular surgery accounted for the largest proportion of trainees (70%, 149/214) and consultants (54%, 175/322). A response rate could not be calculated because the sampling strategy prevented a determination of how many surgeons viewed the invitation to participate.

**Table 1.** Respondent demographic characteristics

Characteristics	Trainee surgeons	Consultant surgeons
	*n* (%)	
Sex		
Female	109 (51)	59 (18)
Male	101 (47)	259 (80)
Prefer not to say	4 (2)	4 (1)
Age (years)		
20–29	55 (26)	
30–39	139 (65)	26 (8)
40–49	17 (8)	122 (38)
50–59		138 (43)
≥60		36 (11)
Prefer not to say	3 (1)	
Trainee grade		
CT1-2, ST1-2	69 (32)	
ST3-4	38 (18)	
ST5-6	52 (24)	
ST7-8	55 (26)	
Region		
East of England	8 (4)	24 (8)
East Midlands	41 (19)	52 (16)
Kent, Surrey and Sussex	1 (1)	6 (2)
London	21 (10)	27 (8)
North East	31 (15)	19 (6)
North West	4 (2)	6 (2)
South West	32 (15)	34 (11)
Thames Valley	1 (1)	6 (2)
Wessex	12 (6)	19 (6)
West Midlands	10 (5)	47 (15)
Yorkshire and Humber	21 (10)	20 (6)
Scotland	11 (5)	34 (11)
Wales	11 (5)	17 (5)
Northern Ireland	3 (1)	6 (2)
No response	7 (3)	5 (2)
Specialty		
Breast	4 (2)	10 (3)
Cardiothoracic	2 (1)	6 (2)
ENT	3 (1)	11 (3)
General	121 (57)	119 (37)
Maxillofacial	1 (1)	5 (2)
Neurosurgery	3 (1)	3 (1)
Ophthalmology	1 (1)	11 (3)
Paediatric	1 (1)	2 (1)
Plastics	9 (4)	7 (2)
Transplant	3 (1)	26 (8)
Trauma and orthopaedics	21 (10)	27 (8)
Urology	8 (4)	14 (4)
Vascular	28 (13)	56 (17)
Other	7 (3)	25 (8)
No response	2 (1)	

Some percentages do not sum to 100% owing to rounding to the nearest whole number.

Mean scores and standard deviations for trainee surgeons and consultant surgeons on each Standard are shown in Table 2. A score of 5 indicates that the Standard is being met. Trainee surgeons reported significantly poorer psychosocial working conditions than consultant surgeons on the *control* (*p* < 0.001, *d* −0.692), *peer support* (*p* = 0.037, *d* −0.184) and *change* (*p* < 0.001, *d* −0.490) Standards.

**Table 2.** Management standards indicator tool scores

Psychosocial hazard domain	Trainee surgeons (*n* = 214)	Consultant surgeons (*n* = 322)			UK benchmarks
	*M* (SD)		*P*	d	*M* (SD)
Demands	3.28^d^ (0.78)	3.18^c^ (0.89)	.158	0.125	3.36 (0.26)
Control	2.93^b^ (0.83)	3.52^e^ (0.86)	<0.001^***^	-0.692	3.43 (0.28)
Managerial support	3.06^b^ (0.87)	3.14^c^ (0.98)	.311	-0.089	3.46 (0.23)
Peer support	3.71^d^ (0.73)	3.86^e^ (0.83)	.037^*^	-0.184	3.77 (0.15)
Relationships	4.06^c^ (0.80)	4.08^c^ (0.97)	.743	-0.029	4.22 (0.61)
Role	3.82^b^ (0.79)	3.86^c^ (0.85)	.611	-0.045	4.08 (0.19)
Change	2.41^a^ (0.81)	2.84^c^ (0.92)	<0.001^***^	-0.490	3.03 (0.28)

UK benchmarks taken from Edwards et al. 2012) [18].

^*^ *P* < 0.05,

^**^ *P* < 0.01,

^***^ *P* < 0.001.

^a^Mean score below the 5th percentile.

^b^Mean score at the 5th percentile.

^c^Mean score at the 10th percentile.

^d^Mean score at the 25th percentile.

^e^Mean score at the 50th percentile.

None of the Standards were met. The *relationships* Standard was closest to being met, with both trainee and consultant surgeons generating a mean score >4, indicating that most respondents did not experience harassment or bullying in the workplace. However, item-level analysis revealed considerable differences by sex among consultant surgeons, with 22% (*n* = 13/59) of female and 10% (*n* = 25/259) of male respondents reporting being often/always ‘subject to harassment in the form of unkind words or behaviour’. Likewise, the proportion of female consultant surgeons that reported being often/always ‘subject to bullying at work’ was double that of males at 14% (*n* = 8/59) versus 7% (*n* = 19/259). Among trainee surgeons, the prevalence of harassment and bullying was lower with no significant sex differences. Nine per cent of male (*n* = 9/101) and 10% (*n* = 11/109) of female trainees reported being often/always harassed, while 3% of both male (*n* = 3/101) and female (*n* = 3/109) trainees reported being often/always bullied.

Poorest performance was observed on the *change* Standard, with both trainee and consultant surgeons generating a mean score <3, indicating a low level of consultation about change and a lack of clarity about how changes will work out in practice. Item-level analysis revealed that far fewer consultants (28%, *n* = 91/322) than trainees (50%, *n* = 108/214) disagreed/strongly disagreed that they had sufficient opportunities to question managers about change at work. The pattern was reversed on the other two change items: 54% (*n* = 174/322) of consultants (trainees, 64%, *n* = 138/214) disagreed/strongly disagreed that staff are always consulted about change at work, and 40% (*n* = 128/322) of consultants (trainees, 48%, *n* = 102/214) disagreed/strongly disagreed that when changes are made at work it is clear how they will work out in practice.

Table 2 also shows mean scores on each Standard compared to UK general workforce benchmarks. The quality of trainees’ psychosocial working conditions fell below the 10th percentile on four Standards (*control*, *managerial support*, *role*, *change*) and below the 50th percentile on the remainder (*demands*, *peer support*, *relationships*). Consultant surgeons’ psychosocial working conditions fell below the 25th percentile on five Standards (*demands*, *managerial support*, *relationships*, *role*, *change*) and were at the 50^th^ percentile on the remaining two Standards (*control, peer support*).

Forty-eight per cent of trainee surgeons (*n* = 103/214) and 38% (*n* = 123/322) of consultant surgeons reported burnout. Bivariate correlations were applied to examine the strength and direction of relations between each Management Standard area and burnout (Table 3). Among trainee and consultant surgeons all seven Standards generated a negative association of at least medium strength (*r* ≥ −0.3), meeting or exceeding the commonly applied (*r* ≥ ± 0.3) threshold for real-world (as opposed to statistical) significance in workplace health and well-being research [24]. Among trainee surgeons, strong negative associations (*r* = ≥ −0.5) were observed between the *demands* and *managerial support* Standards and burnout.

**Table 3.** Intercorrelations between variables

Variable	1	2	3	4	5	6	7	8
1. Demands	(0.77\0.83)	0.41	0.38	0.38	0.40	0.31	0.38	-0.48
2. Control	0.44	(0.82\0.89)	0.64	0.64	0.48	0.60	0.63	−0.49
3. Managerial support	0.47	0.55	(0.87\0.90)	0.67	0.52	0.57	0.71	−0.42
4. Peer support	0.44	0.49	0.67	(0.85\0.87)	0.58	0.49	0.55	−0.42
5. Relationships	0.53	0.42	0.47	0.50	(0.81\0.87)	0.47	0.44	−0.39
6. Role	0.35	0.25	0.44	0.45	0.27	(0.79\0.79)	0.58	−0.35
7. Change	0.39	0.55	0.65	0.41	0.36	0.27	(0.75\0.82)	−0.38
8. Burnout	−0.51	−0.36	−0.52	−0.43	−0.34	−0.30	−0.31	

Trainee surgeons (*n* = 214) below the diagonal, consultant surgeons (*n* = 322) above the diagonal. All correlation coefficients significant at *P* < 0.001. Cronbach’s alpha coefficients on the diagonal.

Regression analyses for trainee surgeons with burnout as the criterion variable are shown in Table 4. The covariates (age, sex, grade) explained 5% of the variance in burnout (Model 1), which was statistically significant (*R*^2^ = 0.046, adjusted *R*^2^ = 0.032, *F*_(3, 205)_ = 3.28, *p* < 0.05). The addition of psychosocial working conditions encompassed in the Management Standards (Model 2) accounted for a significant additional 35% of the variance in burnout (Δ*R*^2^  = 0.351, Δ*F*_(7, 198)_ = 16.493, *p* < 0.001). Thus, the vast majority of explained variance in burnout was accounted for by psychosocial working conditions, with *demands* (*β* = −0.343) and *managerial support* (*β* = −0.336) making significant contributions to the regression model. The predictors together explained a significant 40% of the variance in mental well-being (*R*^2^ = 0.397, adjusted *R*^2^ = 0.367, *F*_(10, 198)_ = 13.05, *p* < 0.001), representing a large effect.

**Table 4.** Hierarchical multiple linear regression analysis for organizational psychosocial hazards predicting burnout, trainee surgeons (*n* = 214)

	Model 1			Model 2		
	*B*	SE *B*	*β*	*B*	SE *B*	*β*
Model 1						
Sex	0.129	0.132	0.067	0.077	0.109	0.040
Age	0.090	0.111	.081	0.062	0.091	0.055
Grade	−0.217	0.085	−0.253^*^	−0.114	0.070	−0.133
Model 2						
Demands				−0.447	0.092	−0.343^***^
Control				−0.064	0.090	−0.053
Managerial support				−0.393	0.109	−0.336^***^
Peer support				−0.110	0.114	−0.079
Relationships				0.025	0.090	.020
Role				−0.008	0.082	−0.007
Change				0.123	0.097	0.098
*R* ^2^	0.046			0.397		
Δ*R*^2^	0.046^*^			0.351^***^		
Adj. *R*^2^	0.032			0.367		

*β*, standardized beta coefficient; *B*, unstandardized regression coefficient; SE *B*, standard error of unstandardized regression coefficient; *R*^2^, explained variance; adj. *R*^2^, explained variance adjusted; Δ*R*^2^, change in explained variance.

^*^ *P* < 0.05,

^**^ *P* < 0.01,

^***^ *p* < 0.001.

Regression analyses for consultant surgeons are shown in Table 5. The covariates (age, sex) explained less than 1% of the variance in burnout (Model 1), which was not statistically significant (*R*^2^ = 0.004, adjusted *R*^2^ = −0.002, *F*_(2, 318)_ = 0.636, *p* > 0.05). The addition of psychosocial working conditions encompassed in the Management Standards (Model 2) accounted for a significant additional 35% of the variance in burnout (Δ*R*^*2*^  = 0.348, Δ*F*_(77, 311)_ = 23.826, *p* < 0.001). Thus, the vast majority of explained variance in burnout was accounted for by psychosocial working conditions, with *demands* (*β* = −0.293) and *control* (*β* = −0.245) making significant contributions to the regression model. The predictors together explained a significant 35% of the variance in mental well-being (*R*^2^ = 0.352, adjusted *R*^2^ = 0.333, *F*_(9, 311)_ = 18.743, *p* < 0.001), representing a large effect.

**Table 5.** Hierarchical multiple linear regression analysis for organizational psychosocial hazards predicting burnout, consultant surgeons (*n* = 322)

	Model 1			Model 2		
	*B*	SE *B*	*β*	*B*	SE *B*	*β*
Model 1						
Sex	0.039	0.118	0.019	−0.124	0.099	−0.059
Age	−0.036	0.035	−0.058	−0.021	0.029	−0.033
Model 2						
Demands				−0.324	0.058	−0.293^***^
Control				−0.279	0.081	−0.245^***^
Managerial support				−0.074	0.077	−0.073
Peer support				−0.055	0.083	−0.047
Relationships				−0.097	0.062	−0.095
Role				−0.012	0.072	−0.010
Change				−0.001	0.076	−0.001
*R* ^2^	0.004			0.352		
Δ*R*^2^	0.004			0.348^***^		
Adj. *R*^2^	−0.002			0.333		

*β*, standardized beta coefficient; *B*, unstandardized regression coefficient; SE *B*, standard error of unstandardized regression coefficient; *R*^*2*^, explained variance; adj. *R*^*2*^, explained variance adjusted; Δ*R*^*2*^, change in explained variance.

^*^ *P* < 0.05,

^**^ *P* < 0.01,

^***^ *p* < 0.001.

## Discussion

This study represents the first assessment of surgeons’ psychosocial working conditions in accordance with the UK HSE’s Management Standards. Psychosocial work environment quality reported by trainees and consultant surgeons fell short of aspirational targets delineated in the Management Standards; trainees reported significantly worse psychosocial working conditions than consultant surgeons on three of the seven Standards. Trainee surgeons’ psychosocial work environment quality across all seven Standards fell below the average for the UK general workforce, while consultant surgeons’ psychosocial working conditions fell below the average on five Standards. Half of trainee surgeons and two-fifths of consultant surgeons reported burnout. The Management Standards made a large contribution to explaining variance in burnout.

Some features of our study need to be considered when interpreting the findings. While social media was effective for participant sampling, it hindered the calculation of a response rate. Response bias may have been present, with surgeons experiencing poor-quality psychosocial working conditions and symptoms of burnout more inclined to participate, leading to their over-representation. Conversely, surgeons experiencing high levels of burnout may be so overwhelmed that they are less likely to make time for survey completion, resulting in their under-representation [25]. Further, surgeons’ psychosocial work environment quality and mental health might be linked to characteristics that we did not measure, such as hospital size and type, that should be accounted for in future research. Finally, the UK workforce benchmark data were collected pre-2012, since which vast changes have been witnessed in the National Health Service and the workplace in general. There is a need for contemporary general workforce and sector- or role-specific benchmark data. The high prevalence of burnout in our sample may partly be attributable to data collection having occurred during the third COVID-19 ‘stay at home’ lockdown order in the first quarter of 2021. Notably, however, these rates are not inconsistent with pre-pandemic UK surgeon burnout studies [26].

One of our starkest findings is that 1 in 5 female and 1 in 10 male consultants reported being often/always harassed at work in the form of unkind words or behaviour. Bullying experienced by female consultants was likewise double the rate reported by male consultants (14% versus 7% often/always bullied). These findings indicate that harassment and bullying are serious problems within UK surgery and highlight the need to develop knowledge on causal factors towards the development of actions to eradicate these unacceptable behaviours.

We found that harassment and bullying were less prevalent among trainees, with 1 in 10 reporting being often/always harassed and 1 in 30 being often/always bullied and no sex differences. These findings appear at odds with that observed in surveys of UK vascular surgery trainees conducted in 2017 and 2021 in which the proportion of respondents reporting having experienced bullying, undermining or harassment rose from 47% to 72% between the two time points [27]. Combining data from the two surveys revealed a large sex difference, with 48% of females and 31% of males reporting having experienced bullying, undermining or harassment during their current placement. The contrasting findings might be attributable to the differing time windows; whereas our study required respondents to consider the previous 6-month period, Madurska and colleagues’ [27] 2017 survey invited respondents to consider their entire trainee experience while the 2021 survey asked respondents to reflect on the previous 4 years. Furthermore, the MSIT that we used to assess harassment and bullying involved a 5-point Likert-style response scale in contrast to a dichotomous yes/no response format. The development of consistent approaches to the measurement of harassment and bullying in surgery would support the generation of reliable estimates on the extent of these phenomena that in turn might stimulate concerted mitigation actions.

A UK-specific evidence base on aspects of a surgical career that are linked to burnout is gradually emerging. Small-scale qualitative research has highlighted the contributory role of a host of organizational and operational (clinical) psychosocial work characteristics [5]. Our study has contributed to this knowledge base by quantifying the quality of surgeons’ organizational psychosocial working conditions in accordance with the UK government’s quality framework and demonstrating quantifiable linkages with burnout. In doing so, the study has corroborated and extended on earlier findings [5] and shone a spotlight on areas where intervention is warranted. The Management Standards approach provides a framework to guide the assessment and reduction of psychosocial risk that ensures legal compliance and signals the priority that organizations place on the well-being of employees. Our findings suggest that risk reduction initiatives involving modification of problematic *job demands* (trainees and consultant surgeons), *managerial support* (trainees) and *control* (consultants) would be particularly helpful in the amelioration of burnout in the surgical population. A qualitative examination of how these problems manifest in the surgical work environment would facilitate the identification of surgery-specific risk reduction strategies. Healthcare organizations that adopt a risk management approach for dealing with psychosocial risk stand to reap the benefits of a healthier and more sustainable surgical workforce.

## Data Sharing Statement

Data are available upon reasonable request from Jonathan.houdmont@nottingham.ac.uk
